# Branched-Chain Amino Acid Supplementation Reduces Oxidative Stress and Prolongs Survival in Rats with Advanced Liver Cirrhosis

**DOI:** 10.1371/journal.pone.0070309

**Published:** 2013-07-25

**Authors:** Motoh Iwasa, Yoshinao Kobayashi, Rumi Mifuji-Moroka, Nagisa Hara, Hirohide Miyachi, Ryosuke Sugimoto, Hideaki Tanaka, Naoki Fujita, Esteban C. Gabazza, Yoshiyuki Takei

**Affiliations:** 1 Department of Gastroenterology and Hepatology, Mie University Graduate School of Medicine, Tsu, Japan; 2 Center for Physical and Mental Health, Mie University Graduate School of Medicine, Tsu, Japan; 3 Division of Dietary Service, Mie University School Hospital, Tsu, Japan; 4 Department of Immunology, Mie University Graduate School of Medicine, Tsu, Japan; CIMA. University of Navarra, Spain

## Abstract

Long-term supplementation with branched-chain amino acids (BCAA) is associated with prolonged survival and decreased frequency of development of hepatocellular carcinoma (HCC) in patients with liver cirrhosis. However, the pharmaceutical mechanism underlying this association is still unclear. We investigated whether continuous BCAA supplementation increases survival rate of rats exposed to a fibrogenic agent and influences the iron accumulation, oxidative stress, fibrosis, and gluconeogenesis in the liver. Further, the effects of BCAA on gluconeogenesis in cultured cells were also investigated. A significant improvement in cumulative survival was observed in BCAA-supplemented rats with advanced cirrhosis compared to untreated rats with cirrhosis (P<0.05). The prolonged survival due to BCAA supplementation was associated with reduction of iron contents, reactive oxygen species production and attenuated fibrosis in the liver. In addition, BCAA ameliorated glucose metabolism by forkhead box protein O1 pathway in the liver. BCAA prolongs survival in cirrhotic rats and this was likely the consequences of reduced iron accumulation, oxidative stress and fibrosis and improved glucose metabolism in the liver.

## Introduction

Branched chain amino acids (BCAA) comprise three essential amino acids: leucine, isoleucine, and valine. BCAA has been used as a supplemental therapy to improve malnutrition in patients with liver cirrhosis [1]. Decreased serum ratio of BCAA to aromatic amino acids is a hallmark of liver cirrhosis: it reduces biosynthesis and secretion of albumin in hepatocytes, and it is also associated with worsened prognosis of cirrhotic patients [1]. Several clinical studies have demonstrated that long-term oral supplementation with BCAA improves the quality of life and event-free survival in cirrhotic patients [2], [3]. In addition, a randomized, controlled trial suggested that BCAA supplementation decreased the incidence of hepatocellular carcinoma (HCC). However, such effect was only evident in obese cirrhotic patients with hepatitis C virus (HCV) infection [4]. Several animal studies have also suggested an anti-hepatocarcinogenic activity of BCAA in obese diabetic mice with insulin resistance [5]. It is therefore possible that BCAA can inhibit hepatocarcinogenesis through amelioration of insulin resistance, because obesity and HCV infection are frequently associated with insulin resistance [6].

Hepatic iron accumulation is observed in a wide variety of conditions including alcoholic or non-alcoholic steatohepatitis and chronic hepatitis C [7], [8]. Diferric iron is highly toxic; it produces oxidative stress by Fenton reaction, which can lead to acceleration of hepatic inflammation, progression of hepatic fibrosis and development of HCC [8]. Furthermore, a previous study performed on chronic hepatitis C patients reported that iron-mediated oxidative stress is associated with a high prevalence of diabetes, and several epidemiological studies on the general population demonstrated that iron overload is a predictive factor for the development of diabetes [9], [10]. BCAA treatment increases the ratio of reduced albumin, which in turn decreases oxidative stress by modulating the redox state of albumin in patients with cirrhosis [11]. This evidence may suggest that BCAA can reduce the iron-mediated oxidative stress through a qualitative alteration of serum albumin.

Nutritional aspects of BCAA on hepatic encephalopathy, liver regeneration or hepatic cachexia have been well documented [12]. However, the pharmaceutical aspect of BCAA in chronic liver disease has yet to be fully validated [1]. The present study therefore investigated whether continuous BCAA supplementation increases cumulative survival of rats with advanced liver cirrhosis and ameliorates the iron accumulation, oxidative stress production and fibrosis in the liver. Further, the effects of physiological and therapeutic range of BCAA concentrations on gluconeogenesis in cultured cells were also investigated.

## Materials and Methods

### Animal Treatment

**Figure 1:**
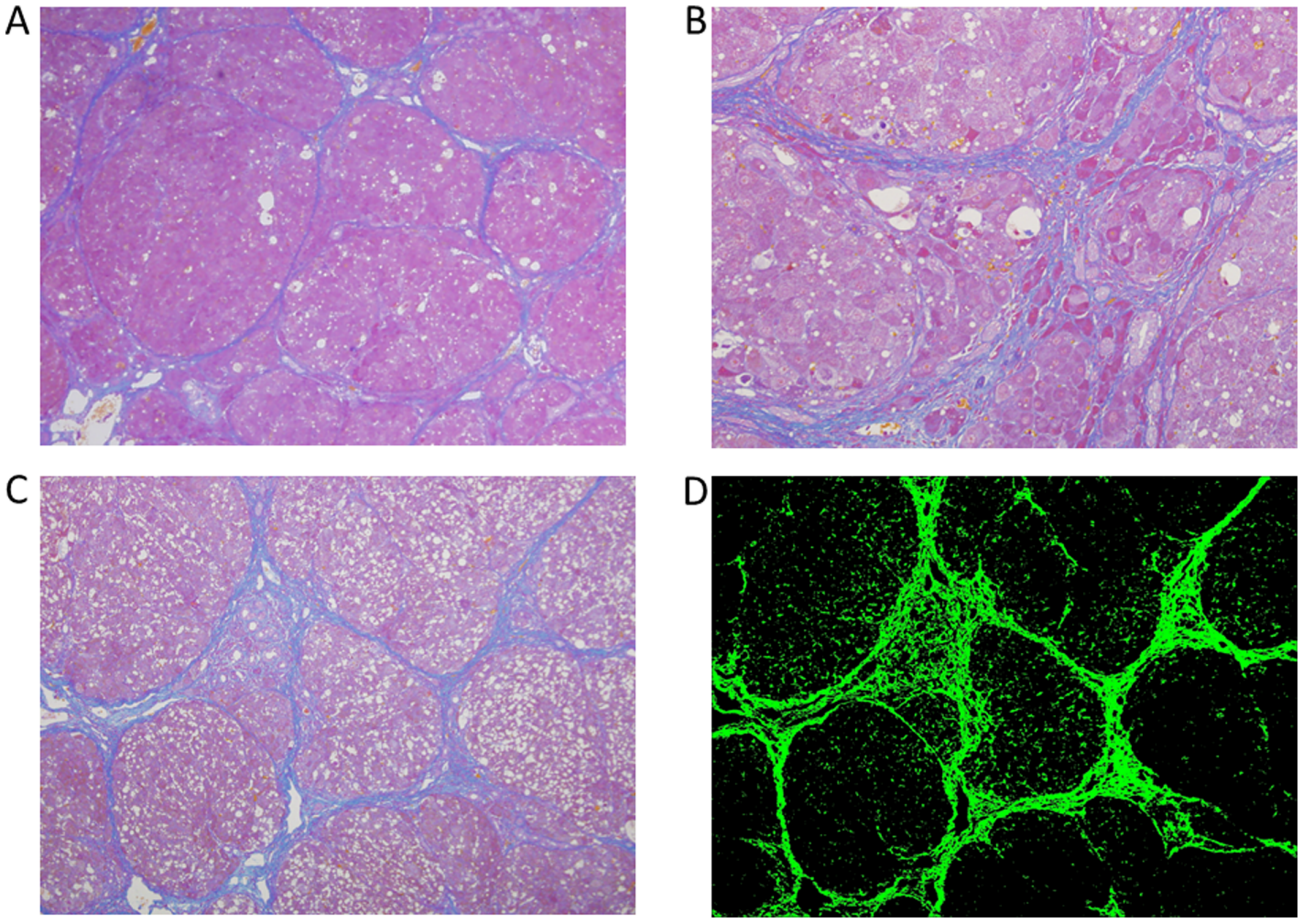
Liver fibrosis in CCl_4_-treated rats. On the 5^th^ week, histological examination of the liver was performed in three rats, and fibrosis consistent with liver cirrhosis was found in all rats (Fig. 1-A, magnification×40). Liver histology on the 21^st^ week showed liver cirrhosis in control rats (Fig. 1-B, magnification×40). Liver histology on the 21^st^ week showed liver cirrhosis in BCAA-treated rats (Fig. 1-C, magnification×40). Azan-positive area in the sample was identified as green color using the Lumina Vision digital analyzing system. The picture in (Fig. 1-D) was derived from (Fig. 1-C). The green and total areas were digitally measured. CCl_4_, carbon tetrachloride.

Male Wistar rats of 6- to 7-week-old were obtained from Charles River Japan (Yokohama, Japan). They were allowed free access to water and standard diet and housed for several days prior to experiments. Chronic liver disease was induced by oral administration of carbon tetrachloride (CCl_4_) (Wako Pure Chem., Osaka, Japan). The rats were given oral 50% CCl_4_ mixed with corn oil at a dose of 1.0 ml/kg of body weight by gavage administration twice a week for 5 weeks and then oral CCl_4_ was reduced to 0.5 ml/kg of body weight twice a week and continued in each rat for another 16 weeks. On the 5^th^ week, histological examinations of the liver were performed in three rats, and fibrosis consistent with liver cirrhosis was found in all rats (Fig. 1-A). On the 5^th^ week, rats were also randomly assigned to treatment or control groups, and fed either with BCAA mixture (Aminoleban EN®, Otsuka Pharm., Tokyo, Japan) 10 ml/kg of body weight/day (BCAA group, n = 9) or saline (control group, n = 12) by gavage administration every day and maintained for 16 weeks. At 6 h after BCAA or saline treatment, oral CCl_4_ was administered by gavage twice a week. The BCAA mixture used in this study had a weight ratio of 1: 2.3: 1.2 for isoleucine: leucine: valine, Fisher ratio 38, 200 kcal/200 ml, protein 13.5 g/200 ml and fat 3.5 g/200 ml. The dose of BCAA was based on our preliminary results and a previous study. Rats were given *ad libitum* access to the diet and drinking water. Dietary intake and body weight were measured every day during the experimental period. Blood was drawn from the tail vein every 4 weeks and centrifuged to separate serum. On the 21^st^ week, rats were sacrificed by overdose of anesthesia and liver samples were rapidly taken. Serum and liver samples were stored at −80°C until analysis. Liver cirrhosis was also found on the 21^st^ week in both control and BCAA-treated groups (Fig. 1-B and 1-C). The experimental protocol of this study was approved by the Hokudo’s Committee on Laboratory Animal Investigation (Permission number HKD10-CAM-01). All procedures were performed under profound anesthesia and all efforts were done to avoid suffering of the experimental animals.

### Measurement of Serum Aminotransferase, Total Bilirubin, Albumin, Glucose and Insulin

Serum concentrations of aspartate aminotransferase, alanine aminotransferase, total bilirubin, albumin and glucose were measured using standard laboratory techniques. Serum insulin levels were measured using the Rat Insulin ELISA KIT (Shibayagi, Gunma, Japan).

### Histology and Immunohistochemistry

Small pieces of each liver were fixed in 10% buffered formaldehyde and then embedded in paraffin. First, Azan staining of paraffin sections was done to assess liver fibrosis. The degree of fibrosis was semi-quantitated using Lumina Vision analyzing system (Mitani Corp., Tokyo, Japan). Briefly, the positive (blue) areas for Azan staining (Fig. 1-C) was identified as green areas by the Lumina Vision software, the background was digitally extracted (Fig. 1-D), and then the green and total area were measured. The results were expressed as % of green/total area. Five liver fields were randomly and blindly selected in each sample and used for analysis.

In addition, sections were incubated with peroxide blocking reagent for 10 min, rinsed with phosphate buffer and again incubated with power block solution for 10 min. Non-specific binding was minimized by leaving the sections in 3% bovine serum albumin in phosphate buffered saline for 30 min. Sections were incubated for 1 h with 1∶100 dilution of anti 4-hydroxynonenal (4-Hne) antibody (Cell Biolabs, Inc., San Diego, CA USA). The sections were rinsed well with phosphate buffer and incubated with super enhancer reagent for 30 min. After rinsing with phosphate buffer, the samples were incubated in super sensitive polymer-horseradish peroxidase immunohistochemistry detection system. After washing thoroughly with phosphate buffer, the samples were incubated in diaminobenzidene substrate solution for 5 min. The sections were counterstained with hematoxylin and observed under light microscopy. Localization and intensity of the staining were assessed simultaneously by two pathologists. The intensity of the staining (4-Hne index) was graded from 0 to 3 as follows: 0, no staining; 1, mild (punctuated labeling); 2, moderate (dense labeling in few cells or in few extracellular foci); 3, strong (dense and homogeneous labeling in many cells or diffuse extracellular staining).

### Measurement of Hepatic 8-hydroxyl-2′-deoxyguanosine (8-OHdG) Concentration

Hepatic total DNA was extracted from liver tissue using a DNA extractor kit (Wako, Osaka, Japan). The hepatic 8-OHdG level was measured using an ELISA kit (High Sensitive 8-OHdG Check; Japan Institute for the Control of Aging, Shizuoka, Japan) after preparations to reduce variability of the enzyme reaction using the 8-OHdG Assay Preparation Reagent Set (Wako, Osaka, Japan). The absorbance of the reaction products were read at 450 nm using a multilabel counter and the data were expressed as ng/ml DNA and after correction by the amount of sample’s DNA level.

### Liver Iron Concentration

Representative samples (approximately 1 g of each rat liver) were collected, weighed and later dried in stove (70°C) to constant weight. Iron concentrations were determined by flame atomic absorption spectrometry. Results were expressed as µmol/g of dry weight.

### Cell Cultures

HepG2 cells were purchased from DS Pharma Biomedical Co. Ltd. (Suita, Japan). The cells were cultured at 37°C in a 5% CO_2_ atmosphere in RPMI1640 medium (Gibco-Life technologies) supplemented with 10% fetal calf serum (FBS, Gibco), penicillin (50 IU/ml) and streptomycin (50 µg/ml). After starvation by culturing with FBS-free medium for 16 h, the medium was replaced to 20 mM of BCAA-containing or BCAA-free RPMI1640 medium [13]. The BCAA mixture (1: 2.3: 1.2 for isoleucine: leucine: valine) was kindly supplied by Ajinomoto Pharm. (Tokyo, Japan). Immediately after medium change, 2.4 mM of diethylmaleate (DEM), a well-known reactive oxygen species (ROS) generator [14], was added to the medium to generate oxidative stress as described previously [15]. The cells were cultured for 2 h in DEM-containing medium.

### RNA Extraction and Quantitative Real-time Polymerase Chain Reaction (PCR)

Total RNA was isolated using Sepazol® RNA II Super (NacalaiTesque, Tokyo, Japan) according to the manufacturer’s protocol and subjected to DNase I digestion (TaKaRa Bio, Otsu, Japan) to eliminate contaminating genomic DNA. First-strand cDNA was synthesized from 5 µg-adjusted total RNA with a SuperScript VILO Master Mix (Life technologies, formerly Applied Biosystem, Foster City, CA). Quantitative TaqMan® PCR was performed with universal TaqMan® Gene Expression Master Mix and commercial primers (TaqMan® Gene Expression assay, Life technologies) for genes *hepcidin* (Rn00584967_m1), transferrin receptor 1 (*Tfr1*, Rn0174701_m1), ferroportin 1 (*Fpn1*, Rn00591187_m1), c-Jun N-terminal kinase (*Jnk*, Rs01453358_m1), forkhead box O1 (*FoxO1*, Rn01494868_m1), phosphoenolpyruvate carboxykinase (*Pepck*, Rn01529014_m1), glucose 6-phosphatase (*G6p*, Rn00689876_m1), mammalian target of rapamycin (*mTor*, Rn00571541_m1) and glyceraldehyde 3-phosphate dehydrogenases (*Gapdh*, Rn9999916_s1). The quantitative PCR was performed in duplicate using 7300 Real Time PCR System (Applied Biosystems-Life technologies). The results were normalized for Gapdh.

### Quantitative Assay of Serum Hepcidin

Serum hepcidin levels were measured using surface-enhanced laser desorption/ionization time of flight mass spectrometry. Serially diluted synthetic hepcidin-25 (Peptide Institute, Osaka, Japan) was used for external mass calibration [16].

### Protein Sample Extraction

Whole protein samples, protein samples of mitochondrial fraction and protein samples of nuclear fraction were extracted using T-PER Tissue ProteoExtraction Reagent Kit (Themo Fisher Scientific, Inc., Rockland, IL USA), NE-PER Nuclear and Cytoplasmic Extraction Reagent (Themo Fisher Scientific) and Mitochondria Isolation Kit (Themo Fisher Scientific) respectively, according to the manufacturer’s instructions. Protein concentration was determined using the Bio-Rad Protein assay (Bio-Rad Laboratories, Inc. Hercules, CA, USA) and BioSpec-mini analyzer (Shimadzu, Kyoto, Japan).

### Western Blotting Analysis

Protein samples were separated using 10% or 12.5% sodium dodecyl sulfate-polyacrylamide gel electrophoresis. The proteins were then transferred to a polyvinylidenedifluoride membrane and blocked with 5% dry non-fat milk or 1% bovine serum albumin in TBST buffer (Tris-buffered saline containing 0.1% Tween 20, pH 7.4) at room temperature for 1 h. The membrane was then incubated overnight at 4°C with primary antibody in TBST, followed by incubation with an alkaline phosphatase-conjugated secondary antibody. The primary antibodies used were anti-copper/zinc superoxide dismutase (Sod1; dilution 1∶1000; ENZO Life Sciences Inc., Farmingdale, NY USA), anti-Mn Sod (Sod2; dilution 1∶1000; ENZO Life Sciences Inc.), anti-Tfr1 (dilution 1∶500, Affinity Bioreagents, Inc., Golden, CO USA), anti Fp1 (dilution 1∶500, Abcam, Tokyo, Japan), anti- phosphorylated c Jun (p-c Jun; dilution 1∶1000, Cell signaling Technology Inc., Danvers, MA USA), anti-phosphorylated JNK (p-JNK; dilution 1∶1000, R&D Systems, Minneapolis, MN USA), anti-FoxO1 (dilution 1∶1000; Cell Signaling Technology Inc.), anti-phosphorylated FoxO1 (p-FoxO1; dilution 1∶1000, Cell signaling Technology Inc.), anti-PEPCK (dilution 1∶400, Santa Cruz Biotechnology Inc., Dallas, TX USA), anti G6P (dilution 1∶1000, Aviva Systems Biology Corp., San Diego, CA USA), and anti-β actin (dilution 1∶4000; BioVision Research Products, Mountain View, CA USA). Thereafter, chemiluminescence development was performed using an Immune-Star chemiluminescent protein detection system (Bio-Rad Laborateries) and, immunoreactive bands were detected and the signal intensities quantified using a Lumino-image analyzer (LAS-4000 mini EPUV, Fuji Film, Tokyo, Japan).

### Statistical Analysis

Data were expressed as mean ± standard deviation. Mann-Whitney U test was used for two-group comparisons. Kaplan-Meier analysis was used for univariate analysis. The difference among three or more groups was performed by analysis of variance using Tukey-Kramer’s test for multiple comparisons. Any p<0.05 was considered to be statistically significant.

## Results

### Improvement of Cumulative Survival Rates in BCAA Group

**Figure 2:**
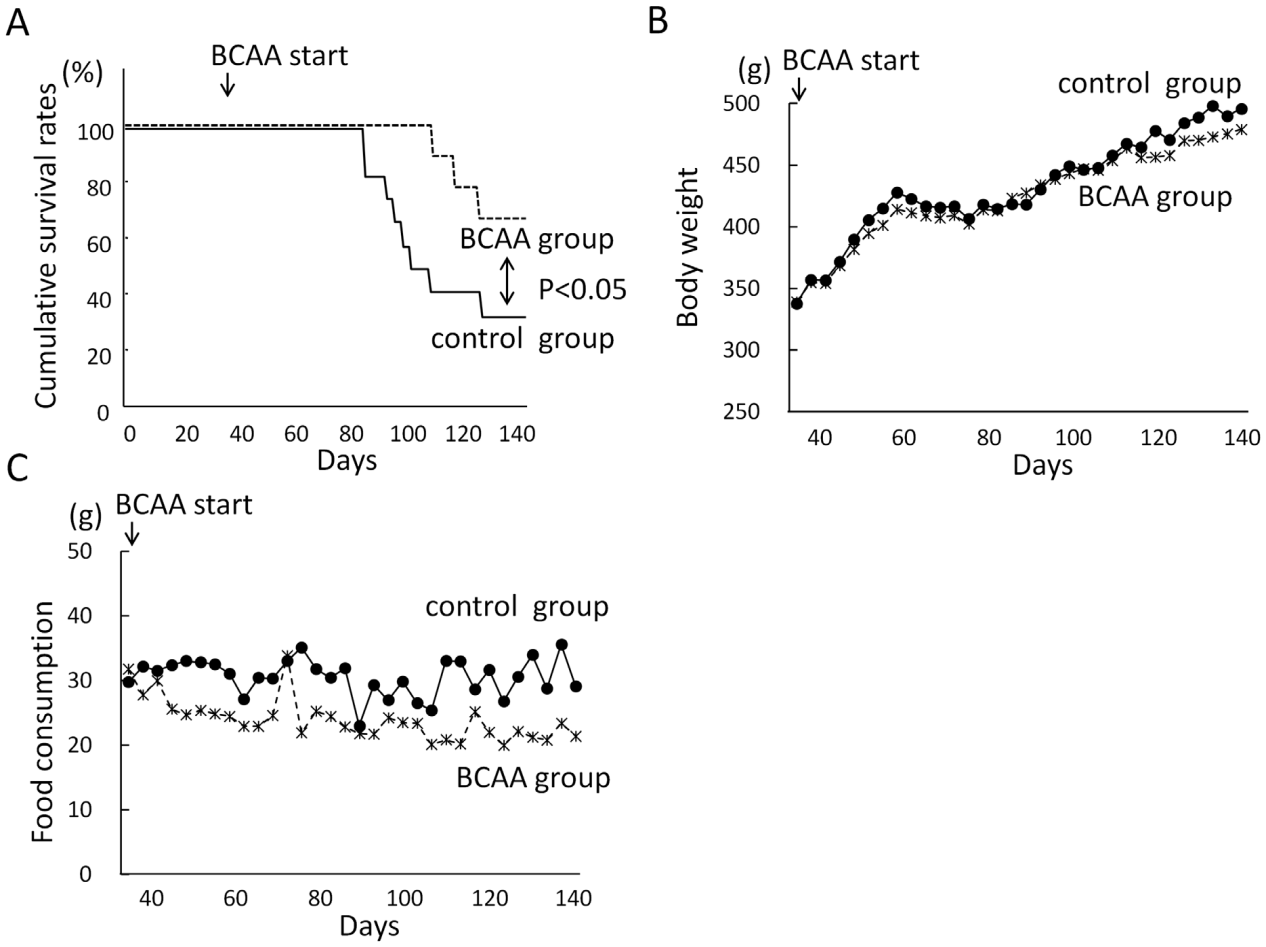
BCAA supplementation prolongs survival of rats with advanced liver cirrhosis. Kaplan-Meier survival curves of rats supplemented or not with BCAA with p values calculated using the log rank test. A significant improvement in survival was observed in BCAA supplemented rats (Fig. 2-A, P<0.05). Body weight of rats supplemented or not with BCAA. There was no significant change in the body weight of rats supplemented or not with BCAA (Fig. 2-B). Food intake of rats supplemented or not with BCAA. Daily food intake was less in the BCAA group than in the control group (Fig. 2-C). BCAA, branched-chain amino acids.

A significant improvement in cumulative survival was observed in rats with advanced liver cirrhosis receiving BCAA supplement versus untreated rats with cirrhosis (Fig. 2-A, P<0.05). While there was no significant change in the body weight of rats supplemented or not with BCAA (Fig. 2-B), daily food intake was less in the BCAA group than in the control group, corresponding to BCAA mixture supplementation (Fig. 2-C).

### Liver Fibrosis and Blood Biochemistry

The degree of fibrosis was 18.6±2.9 and 15.2±1.6% in the control group on the 5^th^ and 21^st^ week, respectively, and 13.7±1.4% in the BCAA group on the 21^st^ week. The fibrosis degree in BCAA-treated group tended to be lower than in the control group.

**Figure 3:**
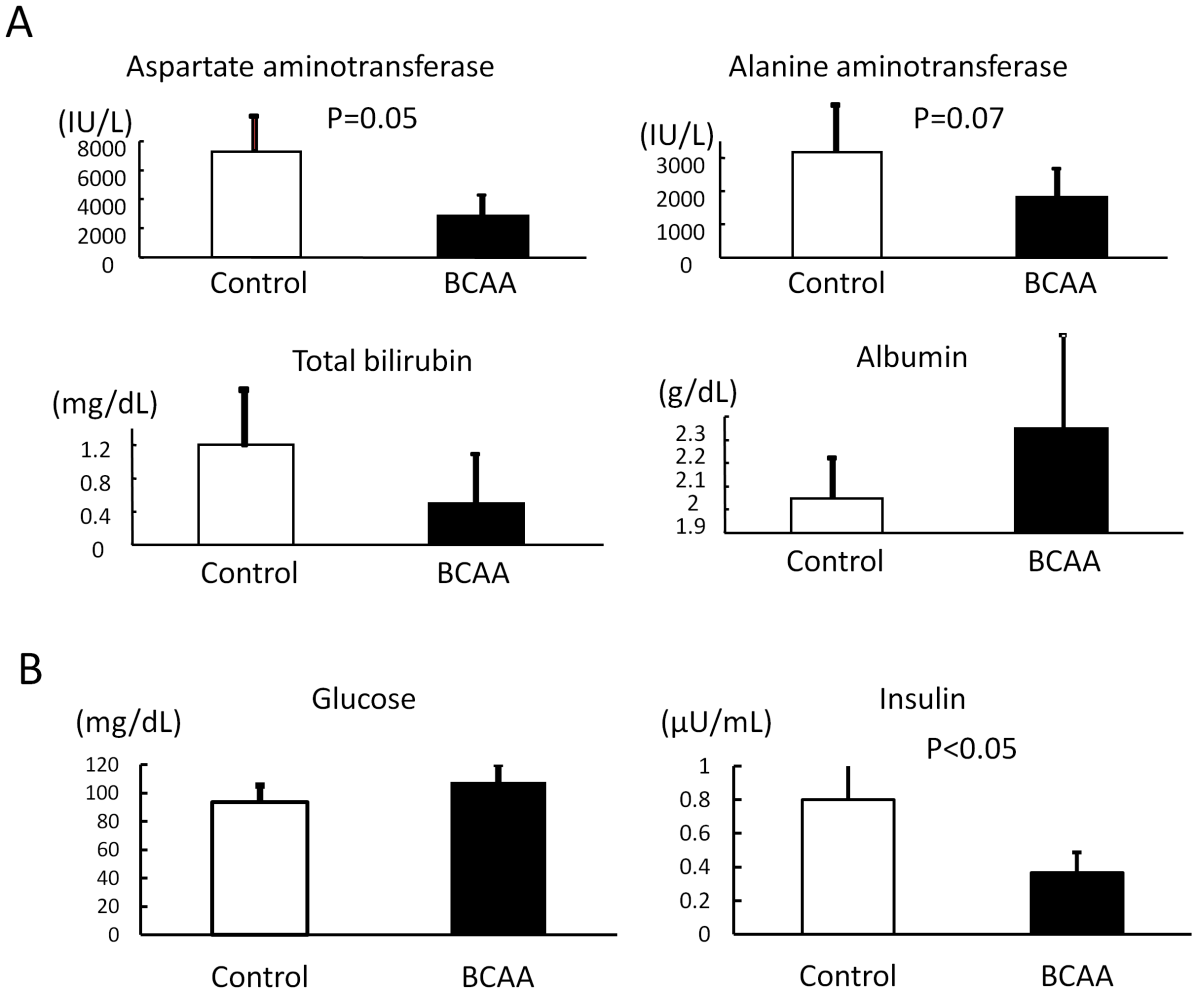
Biochemical data of animals on weeks 17 or 21. Serum aminotransferase activities and total bilirubin levels tended to be lower in rats treated with BCAA (n = 7) than in those without BCAA (n = 5) on weeks 17 (Fig. 3-A). Serum albumin also tended to be higher in BCAA group (n = 7) on weeks 17 (Fig. 3-A). The serum insulin concentration in the BCAA treatment group (n = 6) was lower than in the controls (n = 4) on weeks 21. However, the serum glucose concentration was not differs between groups (Fig. 3-B). BCAA, branched-chain amino acids.

Analysis of blood biochemistry may disclose important signs of hepatic failure such as hyperbilirubinemia and hypoalbuminemia. The mean serum bilirubin levels in the control group were 0.1, 3.7, 1.2 and 0.7 mg/dl and those in BCAA group were 0.1, 1.5, 0.5 and 0.3 mg/dl on weeks 9, 13, 17 and 21, respectively. The mean serum albumin levels in the control group were 2.7, 2.2, 2.0 and 1.8 g/dl and those in BCAA group were 2.7, 2.4, 2.3 and 2.2 mg/dl on weeks 9, 13, 17 and 21, respectively. These results showed a slight increase in the serum bilirubin levels and a marked decrease in the serum albumin levels after repeated CCl_4_ administration in both groups throughout the study. Serum aminotransferase activities and total bilirubin levels were lower in rats with BCAA supplementation than in those without BCAA on weeks 17 and 21, but the differences were not statistically significant. Serum albumin also tended to be higher in BCAA group on weeks 17 and 21 (Fig. 3-A). The serum insulin concentration in BCAA treatment group was lower than in the control group on the 21^st^ week (Fig. 3-B, P<0.05). However, the serum glucose concentration was not different between the groups (Fig. 3-B).

### Immunohistochemistry of 4-Hne, 8-OHdG Concentration and ROS Defense System in the Liver

**Figure 4:**
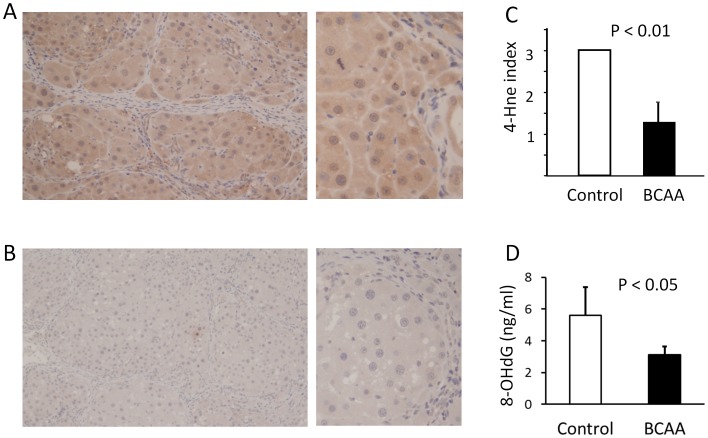
Immunohistochemistry of 4-Hne adducts in rats with advanced liver cirrhosis. A strong intracellular 4-Hne adduct immunostaining (grade 3) is detected in the liver of rats not supplemented with BCAA (Fig. 4-A; Left, magnification×25; Right, magnification×400). A mild intracellular 4-Hne adduct immunostaining (grade 1) is detected in the liver of rats supplemented with BCAA (Fig. 4-B; Left, magnification×25; Right, magnification×400). 4-Hne index is decreased in the BCAA supplementation group (closed columns, n = 6) compared to the control group (open columns, n = 4) (Fig. 4-C). Hepatic 8-OHdG concentration is decreased in the BCAA supplementation group (closed columns, n = 6) compared to the control group (open columns, n = 4) (Fig. 4-D). 4-Hne, 4-hydroxynonenal; BCAA, branched-chain amino acids; 8-OHdG, 8-hydroxy-2′-deoxyguanosine.

4-Hne is a cytotoxic, highly reactive α-, β-unsaturated aldehyde formed by peroxidative metabolism of arachidonic or linoleic acid [17]. Fig. 4-A and 4-B are the representative photomicrographs showing the immunoreactivity for 4-Hne antibodies in liver of rats supplemented or not with BCAA. There was a marked reduction of 4-Hne immunoreactivity in the liver when supplemented with BCAA. 4-Hne index was also decreased in the BCAA supplementation group (Fig. 4-C, P<0.001).

8-OHdG, a modified DNA base product generated by free radicals, is a good biomarker of oxidative DNA damage [8]. Hepatic 8-OHdG level was decreased in the BCAA supplementation group (Fig. 4-D, P<0.05).

**Figure 5:**
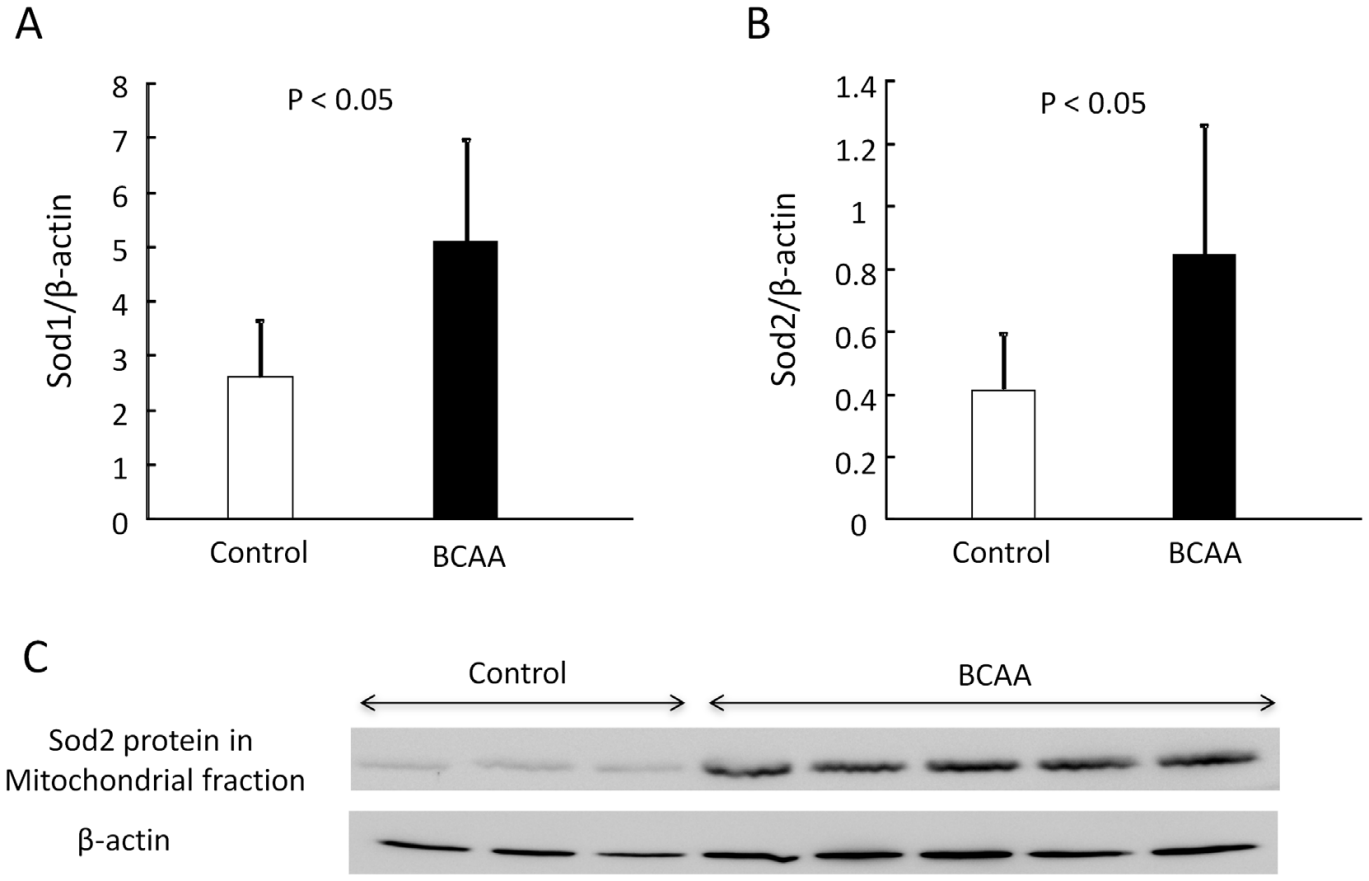
BCAA upregulates ROS defense system in the liver. Copper/zinc Sod (Sod1, Fig. 5-A) and manganese Sod (Sod2, Fig. 5-B) protein levels were increased in the BCAA supplementation group (closed columns, n = 6) compared to the control group (open columns, n = 4). Western blotting of Sod2 protein in mitochondrial fraction is shown in Fig. 5-C. BCAA, branched-chain amino acids; ROS, reactive oxygen species; Sod, superoxide dismutase.

Moreover, the protein levels of the ROS defense system in the liver, including Sod1 and Sod2, were increased by BCAA supplementation (Fig. 5-A, 5-B and 5-C).

### Hepatic Iron Accumulation and Expression of Iron Transporter in Liver

**Figure 6:**
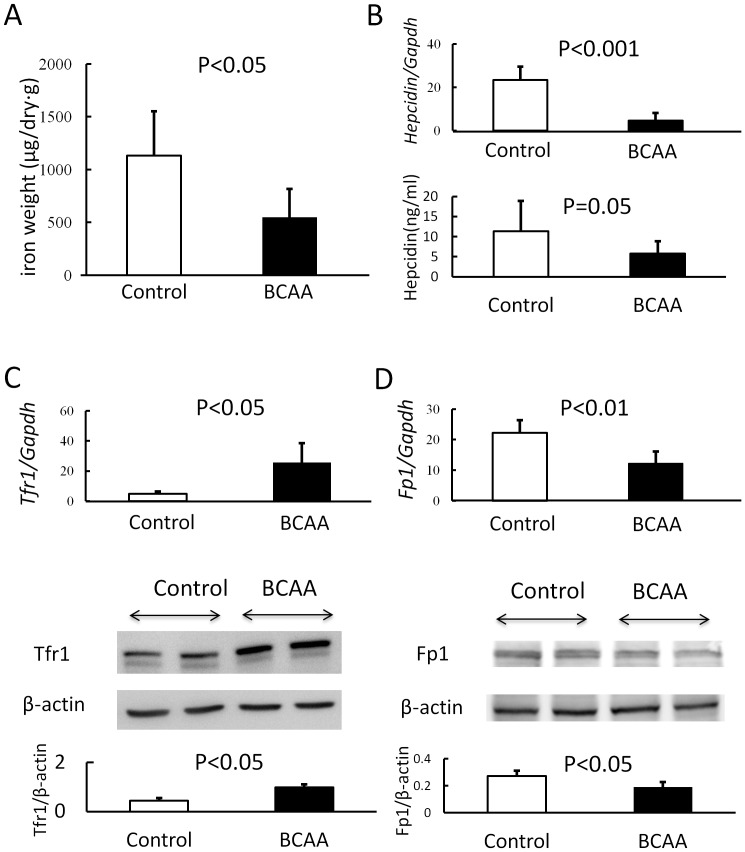
Hepatic iron accumulation and expression of iron transporters in liver. Hepatic iron concentration was measured by atomic absorption spectrometry in rats. Rats fed BCAA mixture (closed columns, n = 6) had lower hepatic iron contents than those fed saline (open columns, n = 4) (P<0.05) on weeks 21 (Fig. 6-A). The gene expression levels of *hepcidin* were measured by real-time PCR and the relative quantities of *hepcidin* mRNA in the liver were normalized to *Gapdh* mRNA. Serum hepcidin was measured by surface-enhanced laser desorption/ionization time of flight mass spectrometry (Fig. 6-B). The gene expression levels of *Tfr1* were measured by real-time PCR and the relative quantities of *Tfr1* mRNA in the liver were normalized to *Gapdh* mRNA. Western blotting of Tfr1 in the liver and the relative quantity of Tfr1 were normalized against beta-actin (Fig. 6-C). The gene expression of *Fp1* was measured by real-time PCR and the relative quantity of *Fp1* mRNA in the liver were normalized against *Gapdh* mRNA. Western blotting of Fp1 in the liver and the relative quantity of Fp1 were normalized against beta-actin (Fig. 6-D). The levels of hepcidin (Fig. 6-B) and Fp1 (Fig. 6-D) were downregulated and those of Tfr1 (Fig. 6-C) were upregulated in the livers of the BCAA group (closed columns, n = 6) on weeks 21. BCAA, branched-chain amino acids; PCR, polymerase chain reaction; Tfr1, transferrin receptor 1; Fp1, ferroportin 1.

Rats fed BCAA mixture by gavage administration had lower hepatic iron contents than those fed saline (Fig. 6-A, P<0.05). Parallel to the improvement in hepatic iron overload, hepatic *hepcidin* mRNA levels were significantly lower in BCAA group as compared to the control group (Fig. 6-B, P<0.001). Serum hepcidin levels tended to be lower in the BCAA group (Fig. 6-B, P = 0.05). We also analyzed the mRNA expression and protein levels of Tfr1 and Fp1, which regulate iron transport in the liver. As shown in Fig. 6-C and 6-D, hepatic Tfr1 mRNA and protein levels were significantly higher in BCAA group, whereas hepatic Fp1 levels were significantly lower.

### Hepatic Gluconeogenesis through FoxO1-dependent Pathway

**Figure 7:**
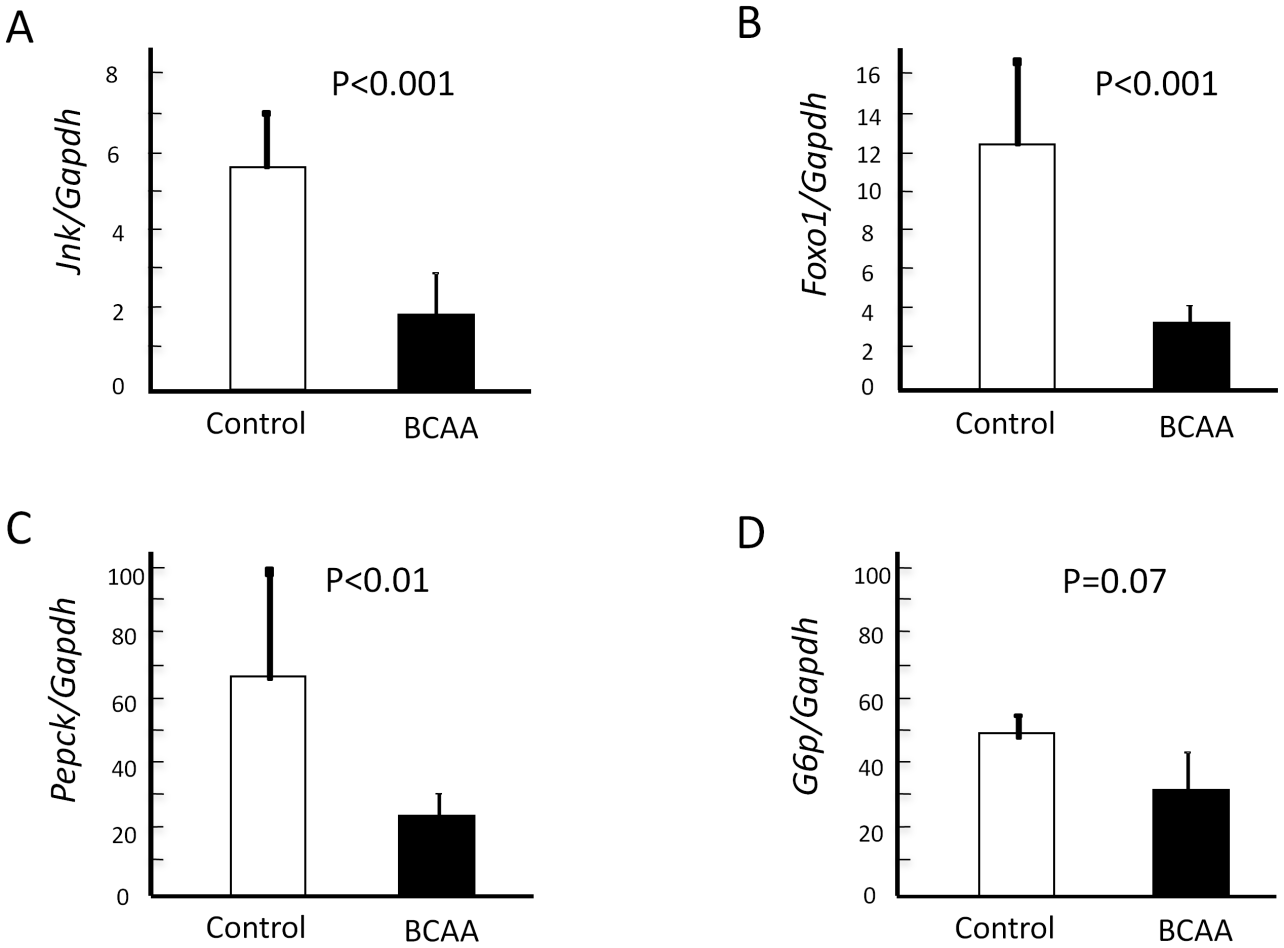
Hepatic gluconeogenesis through FoxO1-dependent pathway. The gene expression of *Jnk* was measured by real-time PCR and the relative quantities of *Jnk* mRNA in the liver were normalized to *Gapdh* mRNA (Fig. 7-A). The expression of *FoxO1* was measured by real-time PCR and the relative quantities of *FoxO1* mRNA in the liver were normalized to *Gapdh* mRNA (Fig. 7-B). The expression of *Pepck* was measured by real-time PCR and the relative quantities of *Pepck* mRNA in the liver were normalized to *Gapdh* mRNA (Fig. 7-C). The expression of *G6p* was measured by real-time PCR and the relative quantities of *G6p* mRNA in the liver were normalized to *Gapdh* mRNA (Fig. 7-D). The expressions of *Jnk* (Fig. 7-A), *FoxO1* (Fig. 7-B), *Pepck* (Fig. 7-C) and *G6p* (Fig. 7-D) were downregulated in the livers of the BCAA group (closed columns, n = 6) compared to the control group (open columns, n = 4). Jnk, c-Jun N-terminal kinase; PCR, polymerase chain reaction; FoxO1, forkhead box O1; Pepck, phosphoenolpyruvate carboxykinase; G6p, glucose 6-phosphatase; BCAA, branched-chain amino acids.

Recent studies demonstrated that a signaling pathway involving the stress-sensitive serine/threonine kinase JNK regulates FoxO1 at multiple levels and that FoxO1 enhances gluconeogenesis through the transcriptional activation of various genes, including PEPCK and G6P [18], [19]. We first analyzed the mRNA expression of *Jnk* and *FoxO1* in rat liver tissues. Rats fed BCAA mixture had lower hepatic *Jnk* and *FoxO1* mRNA levels than those fed saline (Fig. 7-A and 7-B, P<0.01 and P<0.001, respectively). We also examined the expression levels of the rate-limiting enzymes genes in hepatic gluconeogenesis, including *Pepck* and *G6p*. As shown in Fig. 7-C and 7-D, hepatic *Pepck* mRNA levels were significantly lower (P<0.01) and *G6p* tended to be lower in the BCAA group (P = 0.07).

**Figure 8:**
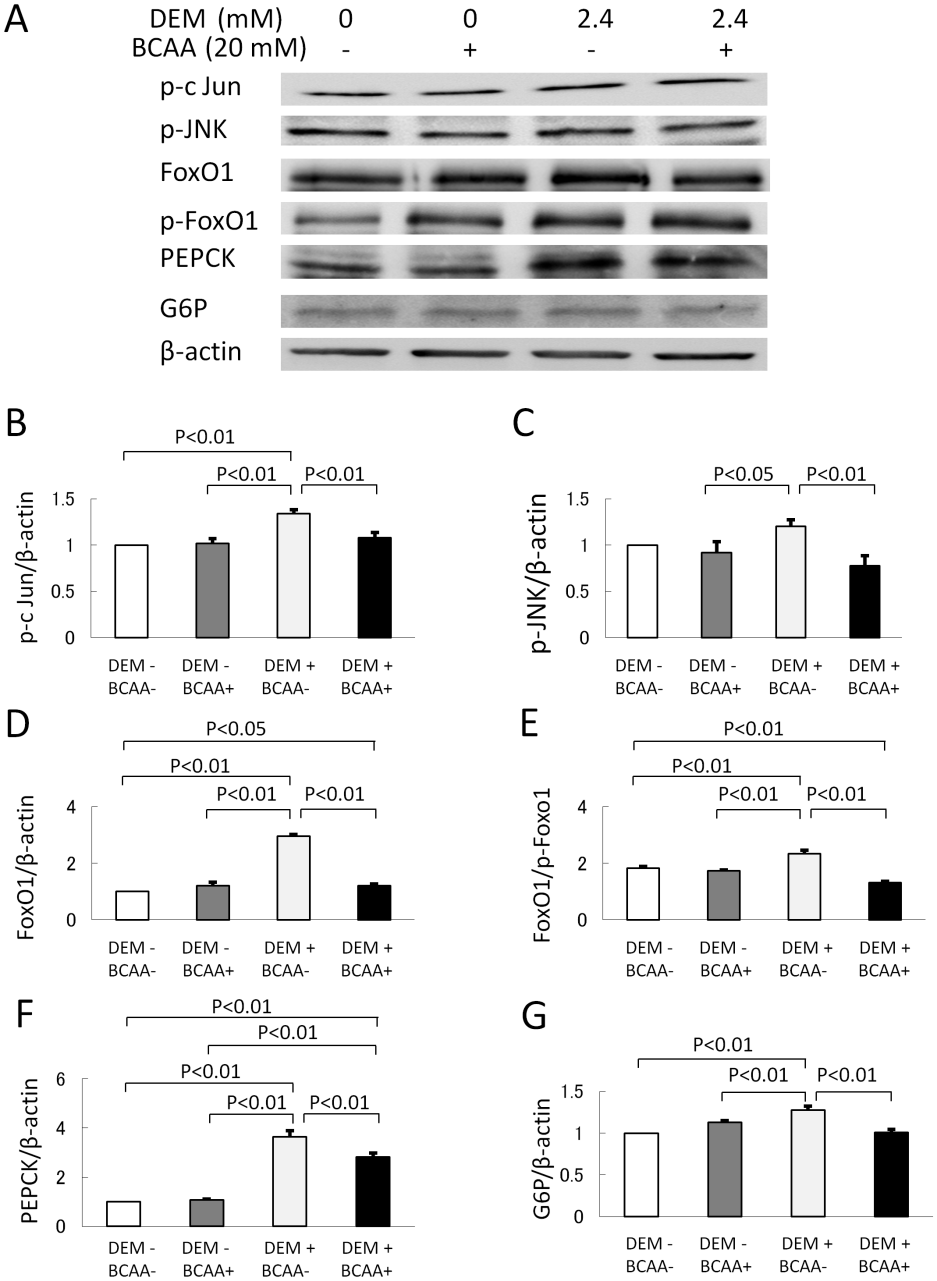
Effect of BCAA on JNK-FoxO1 pathway-mediated gluconeogenesis in cultured cells. Cells were cultured with or without 2.4 mM of DEM for 2 h. Western blotting of p-c Jun, p-JNK and non-phosphorylated FoxO1 in the nuclear fraction, and p-FoxO1, PEPCK, G6P and β-actin in the cytoplasmic fraction of cultured cells (Fig. 8-A). Protein expression level of the p-c Jun was enhanced by DEM (n = 4), but was repressed by BCAA supplementation (n = 4) (Fig. 8-B). Protein expression of p-JNK was enhanced by DEM (n = 4), but it was repressed by BCAA supplementation (n = 4) (Fig. 8-C). Protein expression of FoxO1 was enhanced by DEM (n = 4), but it was repressed by BCAA supplementation (n = 4) (Fig. 8-D). The rate of FoxO1 to p-FoxO1 was elevated by DEM (n = 4), but it was repressed by BCAA supplementation (n = 4) (Fig. 8-E). Protein expression of PEPCK was enhanced by DEM (n = 4), but it was repressed by BCAA supplementation (n = 4) (Fig. 8-F). Protein expression of G6P was enhanced by DEM (n = 4), but it was repressed by BCAA supplementation (n = 4) (Fig. 8-G). DEM, diethylmaleate; BCAA, branched-chain amino acids; p-c Jun, phosphorylated c-Jun, p-JNK, phosphorylated c-Jun N-terminal kinase; FoxO1, forkhead box O1; p-FoxO1, phosphorylated forkhead box O1; PEPCK, phosphoenolpyruvate carboxykinase; G6P, glucose 6-phosphatase.

In vitro experiments using cultured cells showed that the expression of p-c Jun, p-JNK and non-phosphorylated FoxO1 protein in the nuclear fraction of cultured cells was enhanced by DEM, which was repressed by BCAA supplementation (Fig. 8-A, 8-B, 8-C and 8-D). In contrast to the nuclear FoxO1, the expression levels of phosphorylated FoxO1 (p-FoxO1) in cytoplasmic fraction were increased by BCAA supplementation (Fig. 8-A), thereby the ratio of non-phosphorylated FoxO1 to p-FoxO1 is reduced by BCAA (Fig. 8-E). Consequently, the protein expression of PEPCK and G6P was elevated by DEM treatment, but was repressed by BCAA supplement (Fig. 8-A, 8-F and 8-G). FoxO1 protein is known to be distributed in both the nuclear and cytoplasmic fractions. Non-phosphorylated FoxO1 protein in the nucleus activates transcription of various gluconeogenesis genes [18], [19]. Upon phophorylation, FoxO1 protein is exported from the nucleus to the cytosol, resulting in the loss of its transcriptional activity. Gene expression of *PEPCK* and *G6P* is regulated by FoxO1. The present data, therefore, indicate that BCAA can reduce JNK-FoxO1 pathway-mediated glyconeogenesis.

## Discussion

The present study demonstrated that oral supplementation of BCAA prolonged the survival of rats with advanced liver cirrhosis. The survival in diseases that cause liver injury depends on the degree of liver fibrosis and the regenerative ability of remaining hepatocytes. We found that BCAA supplementation attenuated fibrosis in the liver of rats induced by oral administration of CCl_4_. Our present finding is in accordance with a previous report by Kuwahara et al [20]. In addition, this is in line with recent studies showing that long-term oral supplementation with BCAA improves the prognosis in patients with cirrhosis [2], [3]. Interestingly, the prolonged survival due to BCAA supplementation was associated with increased expression of proteins involved in antioxidant defense and reduction of ROS production in the liver. However, previous studies have only documented that BCAA supplementation in cirrhotic patients or rats improves the synthesis, degradation and oxidized/reduced state of albumin [11], [20]. In addition to improving albumin metabolism, we hypothesized that BCAA supplementation decreases ROS production by activating antioxidative mechanisms. Recently, D’Antona et al. showed that BCAA mixture increases the average life span of middle-aged mice and this was associated with upregulation of the ROS defense system genes and reduced ROS production in cardiac and skeletal muscle of mice [21]. Moreover, BCAA have been reported to increase mTORC1 activity, which regulates mitochondrial oxygen consumption and correlates positively with cell oxidative capacity [22]. In the present study, we confirmed that *mTor* expression in the liver is upregulated by BCAA supplementation (data not shown). The data in the presented study demonstrated that BCAA supplementation decreases ROS production in the liver by activating antioxidative mechanisms and by improving albumin metabolism. The prolonged survival due to BCAA supplementation is, at least in part, attributable to reduction of ROS in the liver.

It is well known that increased hepatic glucose production through gluconeogenesis is a major feature of insulin resistance [23]. Using Huh-7.5 cells infected with HCV, Deng L et al. found that HCV increased ROS production and JNK activation, which was directly linked to FoxO1-dependent increase of gluconeogenesis [24]. On the other hand, BCAA are thought to affect glucose metabolism; elevated BCAA and/or loss of BCAA catabolism play an important role in regulating insulin sensitivity [25]. The present study showed that treatment with BCAA is associated with less insulin level without producing a change in the glucose level. Therefore, we hypothesized that ROS can affect gluconeogenesis through FoxO1 pathway and that BCAA ameliorate glucose metabolism by reducing ROS production in the liver. These observations suggest that decreased gluconeogenesis is a result of reduced ROS production in the presence of BCAA, which may lead to amelioration of insulin resistance and contribute to improvement of survival.

It is noteworthy that rats fed BCAA had lower hepatic iron contents than those fed a normal rodent diet. The downregulation in the hepcidin level in the BCAA group accounts for the decrease in hepatic iron content [26]. Moreover, we found that hepatic Tfr1 level was increased and that Fp1 was decreased in the BCAA group. Tfr1 binds to serum transferrin-bound iron and transports it into the cytosol of hepatocytes, whereas Fp1 exports intracellular iron into the sinusoidal blood [27]. Changes in the expression of iron transporters in the present study are probably due to improved hepatic iron overload in BCAA-supplemented rats. We showed that there was a marked reduction in 4-Hne immunoreactivity in the liver of BCAA-supplemented rats. This evidence supports that BCAA supplementation reduces hepatic oxidative stress. Hepatic iron overload plays an important role in the production of ROS [7], [8] and reduction of hepatic iron accumulation by BCAA significantly contributes to reducing oxidative stress in the liver. The precise mechanism of decreased hepatic iron induced by BCAA supplementation is unknown, especially in chronic liver diseases. Further studies will be necessary to identify the pathways by which BCAA ameliorates iron overload.

In conclusion, we showed that BCAA prolongs survival in cirrhotic rats. This was likely the consequence of reduced oxidative stress by reducing iron accumulation, attenuated fibrosis and improved glucose metabolism in the liver of rats. Our study offers a rationale for exploring the beneficial role of BCAA in patients with liver cirrhosis.
